# Regulatory T cells, especially ICOS^+^ FOXP3^+^ regulatory T cells, are increased in the hepatocellular carcinoma microenvironment and predict reduced survival

**DOI:** 10.1038/srep35056

**Published:** 2016-10-11

**Authors:** Jian-Fei Tu, Ya-Hui Ding, Xi-Hui Ying, Fa-Zong Wu, Xin-Mu Zhou, Deng-Ke Zhang, Hai Zou, Jian-Song Ji

**Affiliations:** 1 Department of Radiology and Interventional Radiology, Lishui Central Hospital, Lishui 323000, Zhejiang Province, China; 2 Department of Cardiology, Zhejiang Provincial People’s Hospital, Hangzhou 310000, Zhejiang Province, China; 3 Department of Pathology, Lishui Central Hospital, Lishui 323000, Zhejiang Province, China

## Abstract

Hepatocellular carcinoma (HCC) is a common malignant tumour, especially in Asia. Its prognosis is poor, and there are limited methods for predicting patient survival. This study was carried out to analyse the prognostic value of tumour-infiltrating lymphocytes (TILs), especially regulatory T cells (Tregs), in HCC patients. TILs were analysed in 57 randomly selected HCC patients. The prognostic effects of groups with high and low numbers were evaluated by the Kaplan-Meier and Cox model analyses. Although higher densities of CD3^+^, CD4^+^, and CD8^+^ cytotoxic lymphocytes (CTLs) as well as CD56^+^ NK cells and CD68^+^ macrophages were observed in peritumoural tissue, increased numbers of forkhead/winged helix transcription factor P3^+^ (FOXP3^+^) Tregs were found in intratumoural tissue. Additionally, regarding ICOS^+^ FOXP3^+^ Tregs, an increased prevalence in carcinoma was not only associated with the absolute number but also with the percentage of FOXP3^+^ cells. Higher Treg levels in tumour tissues indicated a worse prognosis, and the FOXP3^+^ Tregs/CD4^+^ T cells ratio was an independent prognostic factor for OS. Therefore, FOXP3^+^ Tregs, especially ICOS^+^ FOXP3^+^ Tregs, contribute to the immunosuppressive HCC microenvironment. High tumour-infiltrating Tregs are thought to be an unfavourable prognostic indicator of HCC.

Hepatocellular carcinoma (HCC) is one of the most common malignant tumours worldwide. A particularly high incidence is found among Asian populations owing to the dissemination of hepatitis virus infections^1^,^2^. Despite remarkably improved diagnostic and treatment strategies, such as surgery, radio frequency ablation and liver transplantation, controlling HCC remains difficult because of its high recurrence rate. Therefore, identification of patients with a high recurrence risk remains a critical clinical issue. HCC is usually present in inflamed fibrotic and/or cirrhotic liver tissue with extensive lymphocyte infiltration due to chronic hepatitis viral infection. Thus, the nature and localization of tumour-infiltrating lymphocytes (TILs) play a central role in the biologic behaviour of HCC^3^,^4^. It is clear that TILs are heterogeneous and contain various immune cell subsets, including innate cells (e.g., NK cells and macrophage) and adaptive immune cells (e.g., CD4^+^ T cells, CD8^+^ T cells, and regulatory T cells (Tregs)), which can suppress or promote the progression of tumours. Therefore, if distinctions are made with respect to lymphocyte type, location, and their combination, a more profound impact on prognosis will be observed compared with only the overall degree of lymphoid infiltration^5^.

As an important subset of functionally immune-suppressive T cells, Tregs play important roles in immunological self-tolerance^6^,^7^. Recently, it has been reported that forkhead/winged helix transcription factor P3 (FOXP3) is the most reliable marker of Tregs^8^. Although mechanisms of suppression by Tregs are still unclear, it has been reported that Tregs can inhibit the function of effector T cells by direct cell-to-cell contact or indirectly via the secretion of immune-suppressive cytokines^9^,^10^. There is accumulating evidence that an increased population of Tregs in TILs or peripheral blood mononuclear cells (PBMCs) is one of the reasons for impaired anti-tumour immunity in cancer-bearing hosts^11^,^12^.

Studies have demonstrated that the proportion of Tregs is increased in TILs and peripheral blood from patients with pancreas/breast adenocarcinoma as well as prostate, ovarian and lung cancers^13^,^14^,^15^. These cells prevent activated CD4^+^ CD25^−^ and CD8^+^ cells from proliferating, which mitigate the immune response against tumour antigens^13^ and associate with tumour prognoses^12^,^16^. These reports suggest that Tregs participate in cancer immune escape and are responsible for difficulties in controlling cancers. Importantly, after patients receive curative resections for gastric cancer, the increased proportion of Tregs is significantly reduced, and the levels are almost equal to those in normal healthy donors^17^. These results strongly suggest that tumour-related factors induce and expand Tregs. There is, however, limited information describing the mechanisms behind Treg accumulation within cancer microenvironments and their expansion locoregionally. Thus, it is important to evaluate the localization of infiltrating Tregs in relation to clinical outcomes.

According to whether or not they express inducible costimulator (ICOS), Tregs can be divided into ICOS^+^ and ICOS^−^ Tregs. *In vitro* experiments have confirmed that the capacity of cytokine production by these two Tregs subsets is different. Whereas the ICOS^+^ Tregs can secrete massive amounts of IL-10 and moderate amounts of TGF-β1, the ICOS^−^ Tregs play a suppressive function mainly through the secretion of TGF-β1^18^. In mice, ICOS^+^ Tregs are characterized by superior survival and highly suppressive properties compared with ICOS^−^ Tregs^19^. Strauss *et al*. reported that ICOS^+^ Tregs are the main suppression subsets in human melanoma^20^.

Huang and Yu confirmed by immunofluorescence that in gastric cancer and papillary thyroid cancer, not only are the ICOS^+^ Treg levels significantly higher in carcinoma tissues than control tissues but the ICOS^+^ Tregs/total Tregs ratios are also higher^21^,^22^. However, the Tregs subsets that participate in tumour immune escape in HCC remains unclear.

In the current study, we investigated the CD3^+^ T cells, CD4^+^ T cells, CD8^+^ T cells, CD68^+^ macrophages, NK cells, and FOXP3^+^ Tregs populations in HCC by immunohistochemistry (IHC), and evaluated the relationship between these findings and clinical outcomes. In the next step, we will focus on ICOS expression on Treg surfaces and analyse the possible mechanisms of Treg recruitment.

## Materials and Methods

### Patients

Tumour and corresponding peritumour tissues (at least 5 cm from the tumour site) were surgically obtained from 57 HCC patients who received a curative resection between 2007 and 2010 at the Hepatobiliary Surgery Department of Lishui Central Hospital. Pathological HCC diagnoses were confirmed by two experienced pathologists via microscopy through standard H&E sections. None of the HCC patients had received immunosuppressive drugs or chemotherapy treatment before surgery. Intratumour tissues were defined as pathologic specimens from HCC tissues. Peritumour tissues were defined as liver tissues at least 5 cm from the primary tumour site. Overall survival was defined as the interval between the dates of surgery and death or the last follow-up. Liver tissues from 20 patients who received partial liver resections for benign disease were used as normal controls. Informed consent was obtained from all patients, and the study was approved by the Ethics Committee of Lishui Central Hospital (Lishui, China). All methods were carried out in accordance with the approved guidelines.

### Immunohistochemistry

Pathological diagnoses were confirmed by standard H&E staining. Paraffin-embedded, 4-lm-thick sections were selected for IHC analysis. Sections were dewaxed and then subjected to heat-induced epitope retrieval with a preheated epitope retrieval solution (10 mM citrate buffer, pH 6.0). Next, endogenous peroxidase activity was blocked, and the sections were incubated overnight with one of the following primary mAbs: mouse anti-CD3, mouse anti-CD4, mouse anti-CD20, mouse anti-CD57, mouse anti-CD68, rabbit anti-CD8 (all were working solutions; from Zhongshan Golden Bridge Biotechnology, Beijing, China), or mouse anti-FOXP3 (1:400; Abcam, Cambridge, UK, ab20034). After incubation with the HRP-conjugated second antibody (Invitrogen, Carlsbad, CA, USA, 11) and development with diaminobenzidine, the sections were counterstained with haematoxylin.

Negative control staining was performed with cold PBS in place of the primary antibody.

### Immunofluorescence

For FOXP3 and ICOS, double-fluorescence immuno-staining was carried out. The first day of the experiment was the same as the paraffin-embedded section IHC analysis, except that the endogenous peroxidase activity was not blocked and the primary mAb was a FOXP3 and ICOS cocktail (both 1:200; Abcam). The next day, sections were incubated with a cocktail of donkey anti-mouse (Alexa Flu or 488, 1:300) and donkey anti-rabbit (Alexa Fluor 594, 1:200) antibodies (both from Invitrogen). Finally, ~30 μL mounting solution antifade^+^ DAPI (Vector 14 Laboratories, Burlingame, CA, USA) was applied and the sections, which were then covered with cover slips. The sections were photographed with a fluorescence microscope (×40 objective lens, ×10 ocular lens; Olympus, Tokyo, Japan) with NIS-Elements software 15.

### Immunohistochemistry double stain

Double staining was performed using the EnVision TMG|2 double stain system (Dako, Glostrup, Denmark) with the following 2 chromogens: DAB^+^ Chromogen (brown colour) for FOXP3 and Permanent Red Chromogen (red colour) for chemokine ligand 20 (CCL20). The primary steps were the same as for the IHC analysis of the paraffin-embedded sections and mouse anti-human FOXP3 (dilution, 1:200) and rabbit anti-human CCL20 (dilution, 1:150) (both from Abcam, Cambridge, UK, ab20034 and ab136904, respectively) were used as the primary antibodies.

### Statistical analysis

Investigators were blinded to the pathological information for each patient at the time of analysis. The histopathologic parameter differences amongst various patient groups were analysed with the Kruskal-Wallis test and the Mann-Whitney two-sample test. Differences in matched tissue samples were determined by the Wilcoxon test for matched pairs. The Kaplan-Meier model was used for OS analysis. The Cox proportional hazards model with stepwise variable selection was used to assess the prognostic contribution of factors in the multi-variable analysis. We considered the results to be statistically significant when P < 0.05. Analysis was performed using SPSS 16.0 software.

## Results

### Patients

The clinicopathologic characteristics of the total patient population are summarized in Table 1. The mean age of the 57 included patients was 50 ± 9 years old with males representing the majority (93%). As for alpha foetal protein (AFP), 44 (77.2%) patients had higher than 200 ng/ml, and the median value was 94.8 ng/ml. Twenty-six (45.6%) patients had hepatitis B virus (HBV) DNA. Before surgery, 45 (79%) patients suffered from abnormal liver function (ALT > 40U/L). The pathology showed that the majority of HCC cases were moderately differentiated or poorly differentiated (54/57, 94.7%). As for tumour size, in 35 cases, the tumours were smaller than 5 cm and in 24 cases, they were bigger than 5 cm. The tumours were counted at the same time, and 29 cases had more than 1 tumour.

### Higher densities of CD3^+^, CD4^+^, and CD8^+^ cytotoxic lymphocytes (CTLs) as well as CD56^+^ NK cells and CD68^+^ macrophages in the peritumoural tissue

**Figure 1:**
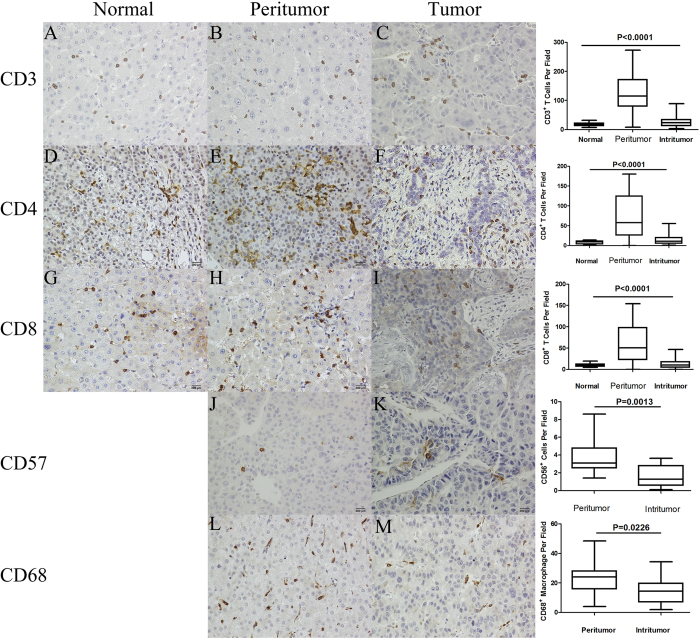
Immunohistochemistry figures of CD3, CD4, CD8, CD57 and CD68.

Much more CD3^+^ T cells, CD4^+^ T cells, and CD8^+^ T cells were found in the peritumoural tissue than in the intratumoural and normal liver tissues (115.3 (range, 8–273.2) *vs*. 23.8 (range, 3.4–89.3) *vs*.18.3 (range, 7.0–32.0), 57.9 (range, 0–180.3) *vs*. 10.8 (range, 1–55.9) *vs*. 9.1 (range, 1.1–14.1), 50.6 (range, 0–154.0) *vs*. 10.4 (range, 4.7–19.4 *vs*. 10.1 (range, 0–46.9); all P < 0.0001) (Table 2). Herein, CD56 and CD68 were used as markers of NK cells and macrophages, respectively. Like the CTLs, compared with the intratumoural tissue, the majority of NK cells and macrophages were also found in the peritumour tissue (3.1 (range, 2.7–4.7) vs. 1.3 (range, 1.0–2.3), 24.1 (range, 17.6–28.5) vs. 14.5 (range, 10–20)) (Fig. 1).

### Increased numbers of FOXP3^+^ Tregs and ICOS^+^ FOXP3^+^ Tregs in the intratumoural tissue

**Figure 2:**
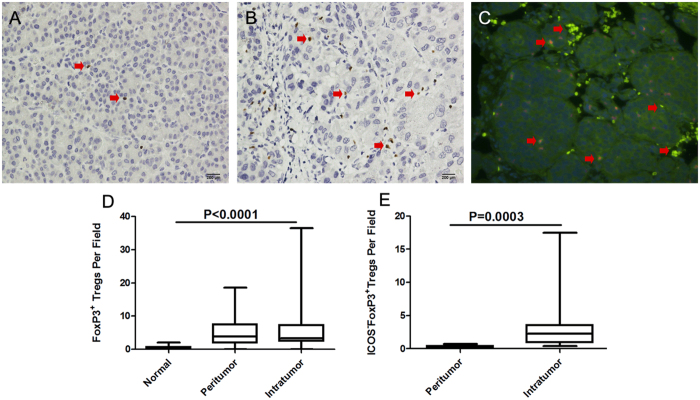
Increased numbers of FOXP3^+^ Tregs and ICOS^+^ FOXP3^+^ Tregs in intratumoral tissue. (**A,B**) Representative immunohistochemistry in normal tissues and intratumoral tissues, the FOXP3^+^ staining was confined to the nucleus (brown). (**C**) Representative double immunofluorescence in intratumoral tissues, the FOXP3^+^ staining was confined to the nucleus (red) and ICOS^+^ was on the cell surface (green). Sections were counterstained with DAPI. (**D,E**) FOXP3^+^ Tregs and ICOS^+^ FOXP3^+^ Tregs in intratumoral tissues were significantly higher. FOXP3 = Forkhead/Winged Helix Transcription Factor P3; Tregs = regulatory T cells; ICOS = inducible costimulator.

Different from the CTLs, the FOXP3^+^ cell density in the intratumoural tissues was close to that of the peritumoural tissue and much higher than in the normal liver tissue (3.9 (range, 0–18.6) vs. 3.3 (range, 0–36.5 *vs.* 0.3 (range, 0–2); P < 0.0001) (Fig. 2A,B,D). However, the number of CD4^+^ T cells in the peritumoural tissue was about six times than in the intratumoural tissue, thus resulting in a significantly higher prevalence of FOXP3^+^ cells (FOXP3^+^ Tregs/CD4^+^ T cells) in the intratumoural tissue (35.6% (range, 37–76%) *vs.* 6.0% (range, 2.0–16%), P < 0.001).

**Figure 3:**
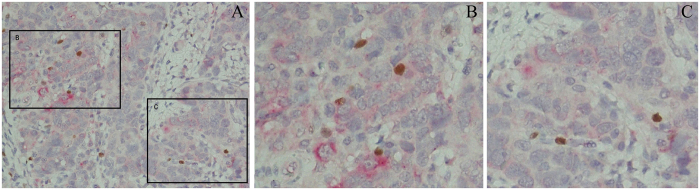
CCL20 tumor-secreted by HCC cells may contribute to the enrichment of Tregs in the tumor tissue. Immunohistochemistry double stain visualized CCL20 in cell cytoplasm (red) and FOXP3 in nucleus (brown). As shown in (**B**,**C**), the main FOXP3^+^ Tregs assemble in CCL20 high-express area and FOXP3^+^ Tregs were much fewer in CCL20 low-express area. CCL20 = chemokine ligand 20; HCC = hepatocellular carcinoma; FOXP3 = Forkhead/Winged Helix Transcription Factor P3.

We further randomly selected 20 samples from 20 patients from the entire cohort to analyse the Treg subsets according to ICOS expression (Fig. 2C). Through double immunofluorescence staining of FOXP3 and ICOS, ICOS^+^ FOXP3^+^ cells (Fig. 3A,B) were observed and preferentially accumulated in the intratumoural tissue (2.3 (range, 0.4–7.2) vs. 0.2 (range, 0.03–0.4), P < 0.001). More importantly, the percentage of ICOS^+^ FOXP3^+^ cells of the total FOXP3^+^ cells was analysed. The ICOS^+^ FOXP3^+^/FOXP3^+^ cell ratio in intratumoural tissue (0.43 (range, 0.28–0.51)) was much higher than that in the peritumoural tissue (0.18 (range, 0.04–0.32), P = 0.0249). These results collectively show the dominant infiltration of ICOS^+^ FOXP3^+^ cells into tumour-affected regions.

### FOXP3^+^ Tregs migration to tumours may be due to increased CCL20 levels

The data above indicated that Tregs (FOXP3^+^) cells were the major players in the tumour immunosuppressive microenvironment, and, more importantly, ICOS^+^ FOXP3^+^ cells were the majority, which was characterized by superior survival and highly suppressive properties. However, little information was available regarding the immune environment favouring their emergence. It is well documented that Tregs express CCR6, the exclusive receptor for CCL20. By IHC double stain, we visualized CCL20 on HCC cells and FOXP3^+^ cells in the same field. As shown in Fig. 3, HCC cells did express higher CCL20 levels than non-tumour cells, and the main FOXP3^+^ Tregs assembled in CCL20 high-expressing area. Our findings provided clues that FOXP3^+^ Tregs migration to tumours may be due to recruitment to CCL20.

### Prognostic Significance of Tregs

According to the ROC curves, the best cutoff values of Tregs, FoxP3^+^ Tregs/CD4^+^ T cells ratio and FoxP3^+^ Tregs/CD8^+^ T cells ratio for the maximum value were 3.2, 0.30 and 0.33 with the sensitivity of 0.741, 0.88 and 0.615, the specificity of 0.667, 0.609 and 0.680 in the respective group.

**Figure 4:**
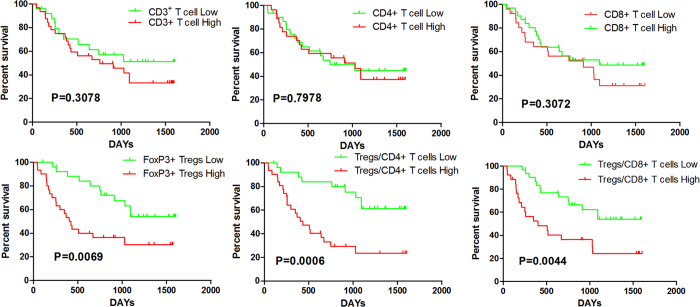
Kaplan-Meier survival curves using the log-rank test to further investigate their association with OS.

In our univariate analysis (Table 3), none of the conventional clinicopathologic features, such as gender, age, AFP, HBV DNA, Child-Pugh grade, or tumour size, could predict the overall survival rate in this study. The patients with more than one tumour had worse prognoses (P = 0.0356). For further analysis, the numbers of CD3^+^ T cells, CD4^+^ T cells, CD8^+^ T cells, and FOXP3^+^ Tregs were investigated the association with OS by using the log-rank test (Fig. 4). As shown in Table 3, the number of CD3^+^ T cells, CD4^+^ T cells, and CD8^+^ T cells in intratumour tissue had no effect on the prognosis of HCC patients. However, patients with high numbers of intratumour infiltrating FOXP3^+^ cells had poorer OS rates than those with lower numbers (P = 0.0069, Table 3). Additionally, patients with higher FOXP3^+^ Tregs/CD4^+^ T cell and FOXP3^+^ Tregs/CD8^+^ T cell ratios also had worse OS rates (P = 0.0006, P = 0.0044, respectively).

Variables that had P values < 0.05 in the univariate analysis were included in a multivariate Cox proportional hazards analysis. We found that intratumour FOXP3^+^ Tregs/CD4^+^ T cell ratios (HR = 3.543; 95% CI: 1.505–8.341; P = 0.004) was independent prognostic factor (Table 3), which indicated that patients with higher FOXP3^+^ Tregs/CD4^+^ T cell ratios were nearly 3.5-fold more likely to die than those with lower ratios.

## Discussion

Immune escape plays a critical role in tumour genesis and progression. The mediators and cellular effectors of the immune system are important components of immunosuppressive tumour environments. Tregs are thought to have indispensable roles in immunological tolerance. Nevertheless, the subsets of Tregs that play a key role in tumour immune escape remain largely undefined. In the present study, increased Tregs in HCC carcinoma tissues were found, which was consistent with previous reports^23^. Moreover, FOXP3^+^ Tregs can be divided into two subsets, ICOS^+^ and ICOS^−^ Tregs, according to their ICOS expression. ICOS^+^ and ICOS^−^ Tregs are regulated by the survival and proliferation of the signalling molecules, ICOS and CD28, respectively, and have different functions. Because of a much higher expression of IL-10, ICOS^+^ Tregs are more suppressive than ICOS^−^ Tregs, and the latter has a high capacity for TGF-β expression^21^,^22^. We quantified ICOS^+^ FOXP3^+^ Tregs in tissues by double immunofluorescence staining. These results showed that HCC patients have an increased prevalence of tumour-infiltrating ICOS^+^ Tregs, not only in terms of absolute number but also in the percentage of total FOXP3^+^ cells. Considering the higher FOXP3^+^ Tregs/CD4^+^ T cell and ICOS^+^ FOXP3^+^⁄FOXP3^+^ cell ratios in intratumoural tissues and higher suppressive capacity, we speculate that ICOS^+^ FOXP3^+^ Tregs may be the main immunosuppressive cell in HCC. Thus, our data expanded and help clarify the concept of Treg-mediated immunosuppression in HCC by demonstrating the predominant role of ICOS^+^ FOXP3^+^ Tregs. To our knowledge, our work represents the first systematic investigation of the frequency of ICOS^+^ FOXP3^+^ Tregs in HCC tissues.

CD3^+^, CD4^+^ T cells and CD3^+^, CD8^+^ T cells are the major components of TILs. Both of them have unique functions in tumour immunity. Therefore, TILs are always considered as an indicator of host immune reactions against cancers because it has been demonstrated that the infiltrating grade of TILs is correlated with a better prognosis in patients with several types of cancer. For HCC, the prognostic value of CTL infiltration remains controversial. In one report, TILs were revealed to have positive prognostic effects^24^, while in other reports, they had no prognostic significance^23^,^25^,^26^. In this study, the number of CD3^+^, CD4^+^ T cells or CD3^+^, CD8^+^ T cells intratumour tissues did not affect the prognosis of HCC patients. Perhaps, the impaired function of intratumour TILs led to these results.

FOXP3^+^ Tregs, which are mostly ICOS^+^ FOXP3^+^ Tregs in HCC tumour tissues, are the main immuno-suppressors in the liver carcinoma microenvironment. As reported in many papers^27^,^28^,^29^, their frequency in intratumour tissues had a negative impact on the overall survival of HCC patients. Moreover, this may be the reason for the higher prevalence of FOXP3^+^ cells (FOXP3^+^ Tregs/CD4^+^ T cells) in intratumoural tissues and their powerful immunosuppression function. Additionally, higher FOXP3^+^ Tregs/CD4^+^ T cell and FOXP3^+^ Tregs/CD8^+^ T cell ratios were also associated with worse prognoses. Our multivariate survival analysis revealed that the FOXP3^+^ Tregs/CD4^+^ T cell ratio was an independent prognostic factor for OS. Patients with a higher intratumoural Treg density had a 3.1 fold higher risk of death than those with a lower FOXP3^+^ Tregs/CD4^+^ T cell ratio. Thus, high-ratio intratumoural Tregs create a generalized immunosuppressive microenvironment and contribute to the ability for tumour cells to escape the host’s immune surveillance^30^. With their immunosuppressive functions, Treg infiltration, and perhaps more importantly, ICOS^+^ FOXP3^+^ Treg infiltration, plays critical roles in tumour progression and clinical behaviour by modifying the host’s immune response.

The high number of FOXP3^+^ Treg tumour-infiltration in the liver carcinoma microenvironment raises the question of their recruitment. There are mainly two possible pathways to explain this result. One of the possible ways is by priming naive CD4^+^ T cells to differentiate into Tregs. As reported in gastric and papillary thyroid cancer, there were clues that plasmacytoid dendritic cells induce Tregs, especially ICOS^+^ Tregs, through the ICOS^−^ICOSL pathway^21^. The other way is that Tregs selectively migrate to tumours, which is mediated mainly by chemokines and their receptors. Recently, accumulating findings showed that CCR6 expression on Tregs also plays a critical role in tumour progression^31^,^32^. Consistent with other research studies, we found that HCC cells not only express CCL^−^20 but also that FOXP3^+^ Tregs assemble in high CCL20 expression areas. In other words, the localization of HCC cells determines the Treg distribution.

In conclusion, our data suggest that Tregs, and not CD3^+^, CD4^+^ T cells, CD3^+^, CD8^+^ T cells, macrophages or NK cells, are significantly enriched in HCC tissues and most were ICOS^+^ FOXP3^+^ Tregs. The higher Treg levels in tumour tissues indicated a worse prognosis and the FOXP3^+^ Tregs/CD4^+^ T cell ratio was an independent prognostic factor for OS. Our study provides at least a clue that FOXP3^+^ Tregs migration to tumours may be due to recruitment to CCL20. If we can deplete or attenuate Tregs by blocking CCL20-CCR6 axis-mediated Treg migration, then it may be a promising novel approach for HCC treatment.

## Additional Information

**How to cite this article**: Tu, J.-F. *et al*. Regulatory T cells, especially ICOS^+^ FOXP3^+^ regulatory T cells, are increased in the hepatocellular carcinoma microenvironment and predict reduced survival. *Sci. Rep.* **6**, 35056; doi: 10.1038/srep35056 (2016).

## Figures and Tables

**Table 1:** Clinicopathologic characteristics of the patients with HCC.

Characteristics	Results
Male/female	53/4
Mean age ± SD, years	50 ± 9
Child-Pugh grade: A/B + C	42/15
Median AFP (IQR), ng/ml	94.8, IQR(10.4, 1629)
HBV DNA: positive/negative	26/31
Median ALT (IQR), U/L	70.5, IQR(43.75, 155.5)
Histologic grade: WD/MD or PD	3/54
Tumor size: ≤5/>5 cm	35/22
Tumor number: single/multiple	29/28

HCC = Hepatocellular Carcinoma; SD = Standard Deviation; AFP = Alpha Fetal Protein; IQR = Interquartile Range; HBV = Hepatitis B Virus; ALT = Alanine Aminotransferase; WD = Well Differentiated; MD = Moderately Differentiated; PD = Poorly Differentiated.

**Table 2:** CD3^+^ T cells, CD4^+^ T cells, CD8^+^ T cells in peritumoral tissue, intratumoral tissues and normal liver tissues.

	Peritumoral tissue	Intratumoral tissue	Normal liver tissue
CD3^+^ T cells	115.3 (range, 8–273.2)	23.8 (range, 3.4–89.3)	18.3 (range, 7.0–32.0)
CD4^+^ T cells	57.9 (range, 0–180.3)	10.8 (range, 1–55.9)	9.1 (range, 1.1–14.1)
CD8^+^ T cells	50.6 (range, 0–154.0)	10.4 (range, 4.7–19.4)	10.1 (range, 0–46.9)

**Table 3:** Univariate and Multivariate analysis of factors associated with overall survival.

Variables	Univariate	Multivariate		
	P-value	HR	95% CI	P-value
Male/female	0.5727			
Age (years)(≤50 vs. >50)	0.3934			
Child-Pugh grade: A/B + C	0.3738			
AFP (ng/ml)(≤400 vs. >400)	0.3795			
HBV DNA: positive/negative	0.8918			
Tumor size: ≤5/>5 cm	0.3198			
Tumor number:single/multiple	0.0356	NA	NA	—
CD3^+^ T cells (≤21 vs. >21)	0.3078			
CD4^+^ T cells (≤11 vs. >11)	0.7978			
CD8^+^ T cells (≤11 vs. >11)	0.3072			
FoxP3^+^ Tregs (≤3.2 vs. >3.2)	0.0069	NA	NA	—
FoxP3^+^ Tregs/CD4^+^ T cells ratio (≤0.30 vs. >0.30)	0.0006	3.543	1.505–8.341	0.004
FoxP3^+^ Tregs/CD8^+^ T cells ratio (≤0.33 vs. >0.33)	0.0044	NA	NA	—

Univariate and multivariate analysis: Cox proportional hazards regression model.

AFP = Alpha Fetal Protein; HBV = Hepatitis B Virus; FOXP3 = Forkhead/Winged Helix Transcription Factor P3; HR = Hazard Ratio; NA = not adopted.
